# Quality of life in persons living with HIV in Burkina Faso: a follow-up over 12 months

**DOI:** 10.1186/s12889-015-2444-4

**Published:** 2015-11-13

**Authors:** Fidèle Bakiono, Patrice Wendpouiré Laurent Guiguimdé, Mahamoudou Sanou, Laurent Ouédraogo, Annie Robert

**Affiliations:** Pôle Epidémiologie et Biostatistique, Institut de Recherche Expérimentale et Clinique (IREC), Faculté de Santé Publique, Université catholique de Louvain, Clos Chapelle-aux-Champs 30, 1200 Brussels, Belgium; Unité de Formation et de Recherche en Sciences de la santé, Université de Ouagadougou, Ouagadougou, Burkina Faso; Institut Régional de Santé Publique de Ouidah, Ouidah, Bénin

**Keywords:** Quality of life, HIV/AIDS, Burkina Faso, WHOQOL HIV-BREF

## Abstract

**Background:**

In Burkina Faso, very little is known about the quality of life of persons living with HIV through their routine follow- up. This study aimed to assess the quality of life of persons living with HIV, and its change over a 1-year period.

**Methods:**

Four hundred and twenty four (424) persons living with HIV were monitored during twelve (12) months from September 2012 to September 2013 in Ouagadougou, the capital city of Burkina Faso. Three interviews were conducted in order to assess the quality of life of patients and its change over time, using the World Health Organization Quality of Life assessment brief scale in patients with Human Immunodeficiency Virus infection (WHOQOL HIV-BREF). The Friedman test was used to assess significant differences in quantitative variables at each of the three follow-up interviews. Groups at baseline, at 6 months and at 12 months were compared using Wilcoxon signed rank test for quantitative data and McNemar test for qualitative variables. Pearson Chi^2^ was used when needed. Multivariable logistic regression models were fit to estimate adjusted odds ratio (OR) and 95 % confidence intervals (95 % CI). Trends in global quality of life score and subgroups (status related to Highly Active Anti Retroviral Treatment (HAART) using univariate repeated measures analysis of variance were assessed. A *p*-value less than 0.05 was considered significant.

**Results:**

At baseline, quality of life scores were highest in the domain of spirituality, religion and personal beliefs (SRPB) and lowest in the environmental domain. This trend was maintained during the 12-month follow-up. The global score increased significantly from the beginning up to the twelfth month of follow-up. Over the 12 months, the baseline factors that were likely to predict an increase in the global quality of life score were: not having support from relatives for medical care (*P = 0.04*), being under HAART *(P = 0.001)*, being self-perceived as healthy (*P = 0.03*), and having a global quality of life score under 77 (*P < 0.001*).

**Conclusions:**

Our findings suggest the need to promote interventions to empower people living with HIV/AIDS through income generating activities. Such activities will enhance the quality of life of persons living with HIV in Burkina Faso. This could focus mostly on treatment-naïve HIV patients, lacking support from relatives and those who perceive themselves as ill.

## Background

Epidemiological studies have shown that early treatment of persons living with HIV/AIDS (PLWHA) using Highly Active Anti-Retroviral Treatment (HAART) substantially reduces their risk of transmitting the virus and/or developing HIV-related illness [1, 2]. HAART has also proven its efficacy in PLWHA by decreasing HIV-associated mortality and morbidity rates [3]. Given the improved life-expectancy of people living with HIV, there is a need to assess their quality of life on a long term basis. In Burkina Faso, UNAIDS reported in 2012 a seroprevalence of 1.1 % in the general population [4]. The same year, 45000 PLWHA were under HAART, which accounted for 45 % of the total estimated number of PLWHA in need of treatment [4]. A previous cross sectional study on the quality of life of PLWHA, with or without treatment, showed that treated individuals were likely to have a better quality of life compared to treatment naïve-infected subjects in Burkina Faso [5]. Another longitudinal study which assessed the quality of life of persons initiating the treatment showed the trends of their quality of life over a 12-month period [6]. However, very little is known about the trend of the quality of life over time in untreated PLWHA compared to treated patients. Exploring quality of life trends in treated and untreated patients will help the health care system develop adapted strategies to better manage the PLWHA in low-income countries.

This study aimed to assess the quality of life of persons living with HIV in Burkina Faso, in their routine follow-up and its change over time.

## Methods

A longitudinal study was conducted from September 2012 to September 2013, in Ouagadougou, capital city of Burkina Faso, in the following five healthcare facilities, chosen for the high number of PLWHA under their care: two (2) public healthcare facilities (under the national healthcare system) and three (3) community-based healthcare organizations. The public healthcare facilities were as follows: the District Hospital of Boulmiougou (DHB) and the University Hospital Yalgado Ouédraogo (UHYO, Day Hospital Unit). The community-based organizations were as follows: the Center for Information, Counseling and Documentation on AIDS and Tuberculosis (CICDoc), the Association African Solidarity (AAS), and the Association Laafi la Viim (ALAVI). The PLWHA followed up by these five (5) facilities accounted for 35.03 % of the cohort followed up nationwide.

Based on a 95 % confidence level, assuming that 50 % of PLWHA should experience an increase in their quality of life during the 12-month follow-up, with 5 % precision, the required sample size was 385. In order to account for the possibility of patients who will not attend the follow-up interviews, this sample size was increased by 10 %, giving a total sample size rounded up to 424 PLWHA. The number of interviewees per structure was determined according to the size of the structure. The numbers were 150, 135, 64, 39 and 36, respectively for the DHB, the Day Hospital Unit, the CICDoc, the AAS and the ALAVI. In each facility, recruitment was done through the daily routine check-up, using a systematic sampling technique. Selected patients were then followed for 12 months. To assess the quality of life, the World Health Organization’s Quality of Life HIV brief scale [WHOQOL HIV- BREF] [7], the short version of the World Health Organization’s Quality of Life HIV [WHOQOL HIV] [8] was used. The following reasons justified the choice of this scale: 1) the scale is specific to PLWHA; 2) by its length (31 questions), the WHOQOL HIV-BREF is well suited for investigation and for routine consultation; 3) French is the official language in Burkina Faso and there is a validated French version of the WHOQOL HIV-BREF [9]; 4) Moore is the national language spoken by the majority of Burkinabe [10], and a Moore version of WHOQOL HIV-BREF with good psychometric properties is available [11]; and 5) unlike most quality of life assessment scales, which are usually developed in the western world, the WHOQOL HIV and its short version were developed in centers around the world, including two centers in Africa, making them cross-culturally sensitive [12]. The reliability of the scale used in our study was assessed through the internal consistency using the Cronbach’s alpha.

The WHOQOL HIV- BREF is a scale with 31 questions asking the respondent to assess his/her own quality of life in various ways during the 2 weeks preceding the survey. It explores six domains of the quality of life (physical, psychological, level of independence, social relationships, environment and spirituality, religion, and personal beliefs (SRPB)). Each domain explores a certain number of facets of daily life. The physical domain refers to pain and discomfort, energy and fatigue, sleep and rest, as well as symptoms of HIV. The psychological domain includes positive feelings thinking, learning memory and concentration, self-esteem, body image and appearance, and negative feelings. As for the level of independence domain, it explores mobility, activities of daily living, dependence on medication or treatments, and work capacity. The social relationships domain covers personal relationships, social support, sexual activity and social inclusion. The environment domain refers to physical safety and security, home environment, financial resources, health and social care (accessibility and quality), opportunities for acquiring new information and skills, participation in and opportunities for recreation/leisure activities, physical environment (pollution, noise, traffic, climate), and transport. Finally, the spirituality, religion and personal beliefs domain includes spirituality, religion and personal beliefs, forgiveness and blame; concerns about the future; as well as death and dying.

Answers to the questions were rated on a Likert scale from 1 (very negative) to 5 (very positive). Twenty-nine questions were used to construct a score per domain and an global quality of life score. The score of a specific domain was obtained by multiplying the average score of the domain by 4, leading to scores ranging from 4 to 20. One question explores quality of life in general and another one explores general health. The global quality of life score is obtained by summing scores of domains, leading to scores ranging from 24 to 120. Higher scores indicate better quality of life in the domain or better quality of life overall.

Data were collected through 3 interviews by well-trained interviewers. The first interview was conducted at the beginning of the study; the second was conducted at 6 months and the third was conducted at 12 months after the baseline interview. All the 424 PLWHA answered the questionnaire at the first interview. Only 384 answered at the second interview and 351 answered at the third interview. Among people who did not answer the third interview, one refused to continue the study at the second interview and two died. Eleven patients who had not attended the second interview came to the third. These 11 were excluded from matched analyses.

Analyses were performed using IBM SPSS 21.0. The Friedman test was used to assess significant differences in quantitative variables at each of the three follow-up interviews. Groups at baseline, at 6 months and at 12 months were compared using Wilcoxon signed rank test for quantitative data and McNemar test for qualitative variables. Pearson Chi^2^ was used when needed. Multivariable logistic regression models were fit to estimate adjusted odds ratio (OR) and 95 % confidence intervals (95 % CI). A *p*-value less than 0.05 was considered significant. We assessed trends in the global quality of life score and subgroups (status related to HAART) using univariate repeated measures analysis of variance. The present study was approved by the Ethics Committee for Health Research from the Ministry of Health of Burkina Faso. All participants who were contacted agreed to participate in the study and signed a free and informed consent.

## Results

Four hundred and twenty four (424) individuals agreed to participate in the study. At baseline, 12.5 % were male. 67.2 and 32.8 % of participants were respectively recruited in public healthcare facilities and community-based facilities. Over 40 % (44.8 %) of patients were living with another person and 27.1 % were treatment-naive HIV subjects. Fewer than 70 % (67.9 %) of patients had undergone treatment for at least 1 year. The mean duration under treatment was 5 ± 3.1 years. Approximately 1/3 of the respondents did not attend school. Just over half of the patients (50.7 %) worked in small trading or in the informal sector. Over 80 % (81.4 %) of the respondents had shared their HIV status with a family member.

Out of the 424 people at baseline, 351 took part in the last interview (month 12). When comparing the characteristics of respondents who participated in the last interview and those who did not, we did not find significant differences, apart from HAART status. Nevertheless, the proportion of respondents not yet under HAART, who participated in the third interview, was lower (23.8 %) than the proportion of the respondents not yet under HAART who did not participate in the third interview (37 %) (Table 1).

**Table 1:** Baseline characteristics of the participants of the all study compared to those who didn’t attend the third interview

Characteristics at baseline	Third interview participants (*n* = 351^a^)	Participants who didn’t attend 3rd interview (*n* = 73^a^)	*P-value*
	Mean ± SD	Mean ± SD	
	%	%	
Age (mean) in years	37.5 ± 8.2	37.8 ± 10.0	0.81
Gender			0.99
Male	12.5	12.5	
Female	87.5	87.5	
Marital status			0.94
Alone	55.3	54.8	
In couple	44.7	45.2	
Matrimonial system			0.41
Monogamus	75.4	68.6	
Polygamous	24.6	31.4	
Level of education			0.57
Illiterate	32.2	35.6	
Literate	67.8	64.4	
Religion			0.42
Muslims	44.3	37.0	
Catholics	41.1	49.6	
Protestants	14.6	13.7	
Profession			0.03
Public, private employee	16.2	15.1	
Trade & informal sector	52.4	42.5	
Housewives	23.6	23.3	
Farmers	2.0	8.2	
Students	2.3	5.5	
Jobless	3.4	5.5	
HIV services provider			0.79
Hospital	67.0	68.5	
Community-based	33.0	31.5	
HAART status			0.01
Under treatment	76.2	63.0	
No treatment	23.8	37.0	
Sexual behavior			0.36
Risky	38.5	44.3	
No risk	61.5	55.7	

^a^Total may differ according to characteristics

**Fig. 1:**
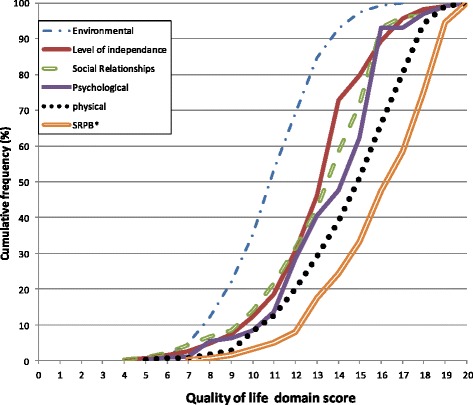
Cumulative frequency polygon of quality of life domain scores at baseline. *SRPB: Spirituality, Religion and Personal Believes

**Fig. 2:**
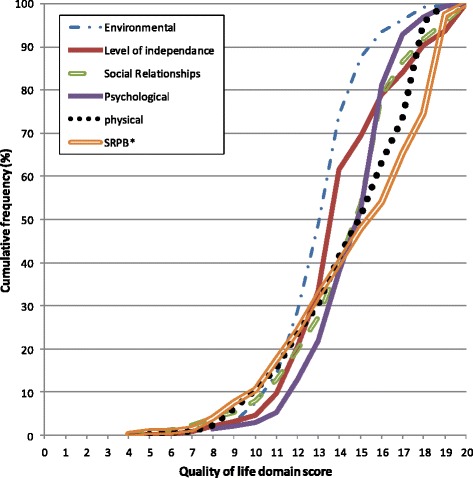
Cumulative frequency polygon of quality of life domain scores at month 12. *SRPB: Spirituality, Religion and Personal Believes

The assessment of the reliability of the whole scale at baseline gave a Cronbach’s alpha set at 0.85. At baseline, the highest scores of quality of life were recorded in the domain of spirituality, religion and personal beliefs (SRPB) and the lowest scores were in the environment domain. This trend was maintained during the 12-month follow-up (Figs. 1 & 2).

From the beginning of the follow-up to the sixth month, four domains increased significantly (Table 2): the psychological domain (+0.7), level of independence (+2.2), social relationships (+1.4) and the environmental domain (+2.6). The global quality of life score also significantly increased from baseline to month 6 (from 82.9 ± 10.9 to 84.6 ± 10.2). When comparing the domain scores from baseline to the twelfth month of the follow-up, the following domains showed significant progress: the psychological domain (+0.8), level of independence (+1), social relationships (+1.2) and the environmental domain (+2.1). The global score not only increased significantly from baseline to the twelfth month (from 82.9 ± 10.9 to 87.0 ± 9.1), but also from the sixth month to the twelfth month (from 84.6 ± 10.2 to 87.0 ± 9.1).

**Table 2:** Changes in quality of life’s domains scores over 12 months in persons living with HIV in Burkina Faso (*n* = 340)

Quality of life’s domains	Interview 1 (Baseline)	Interview 2 (Month 6)	Interview 3 (Month 12)
	mean ± SD	mean ± SD	mean ± SD
Physical	14.9 ± 2.7	13.1 ± 2.6^a^	14.8 ± 3.0^b^
Psychological	13.7 ± 2.7	14.4 ± 2.5^a^	14.5 ± 2.0^a^
Level of independence	13.4 ± 2.5	15.6 ± 3.2^a^	14.4 ± 2.7^b a^
Social relationships	13.5 ± 2.8	14.9 ± 3.5^a^	14.7 ± 2.8^a^
Environmental	11.0 ± 2.2	13.6 ± 2.7^a^	13.1 ± 1.9^b a^
Spirituality	16.3 ± 2.6	13.3 ± 3.1^a^	15.2 ± 3.5^b a^
Global score of quality of life	82.9 ± 10.9	84.6 ± 10.2^a^	87.0 ± 9.1^b a^

^a^Compared with scores at baseline, Wilcoxon test (*p* < 0.05)

^b^Compared with scores at Month 6, Wilcoxon test (*p* < 0.05)

**Fig. 3:**
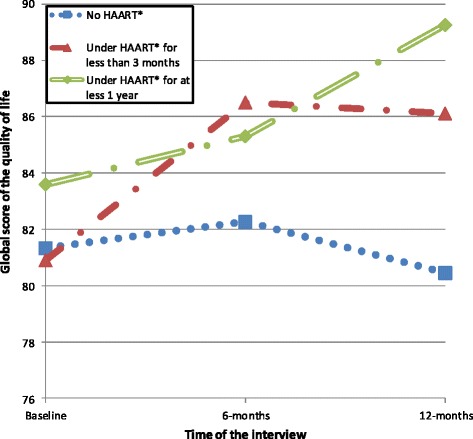
Trends of global score of quality of life over 12 months by treatment group. *HAART: Highly Active Anti Retroviral Treatment

Regarding HAART status, consistently high global quality of life score in patients under treatment for at least 1 year was observed (Fig. 3); they started with the best scores (83.6) and they ended the follow-up with the best scores (89.2). Those not yet under treatment showed a trend of a consistently low global quality of life score in the 12-month follow-up; they started with the second best scores (81.3) and they ended the follow-up with the worst scores (80.4). As for patients under treatment for less than 3 months at baseline, their global quality of life score leapt between recruitment and the sixth month of the follow-up (from 80.9 to 86.5) before stabilizing (86.1 at the end of the follow-up). Starting with the lowest score (80.9), patients under treatment for less than 3 months at baseline ended the follow-up with the second best scores (86.1), while those not under treatment started with the second best scores (81.3) and ended with the worst scores (80.4).

When considering the global quality of life score according to PLWHA characteristics, significant increases were recorded, except in men and people with an AIDS status. In people not yet under treatment, a decreased, yet non-significant, of global quality of life score was recorded (Table 3). Using univariate repeated measures analysis of variance, a linear trend was observed over time and the HAART status significantly affected the global quality of life score over time (F = 8.04; *p* < 0.001).

**Table 3:** The global score of quality of life at baseline and at month 12: comparison based on the characteristics of persons living with HIV in Ouagadougou, Burkina Faso

Variables at baseline	Global score of Quality of life at baseline	Global score of Quality of life at Month 12	*p-value* ^a^
	Mean ± SD	Mean ± SD	
Gender			
Male (*n* = 44)	87.4 ± 9.4	88.3 ± 10.8	*0.66*
Female (*n* = 307)	82.2 ± 10.8	87.1 ± 8.9	*<0.001*
HAART started			
Before 2005 (*n* = 52)	82.4 ± 10.4	90.4 ± 6.9	*<0.001*
2005 and later (*n* = 209)	83.7 ± 11.0	88.9 ± 7.9	*<0.001*
Education			
No school (*n* = 113)	79.6 ± 10.3	86.0 ± 9.5	*<0.001*
School (*n* = 238)	84.4 ± 10.6	87.8 ± 8.9	*<0.001*
Self perceived as healthy			
No (*n* = 204)	85.4 ± 9.6	87.6 ± 8.3	*0.021*
Yes (*n* = 115)	78.7 ± 11.3	86.5 ± 10.0	*<0.001*
Serology status			
Asymptomatic (*n* = 268)	84.0 ± 10.3	87.3 ± 10.3	*0.02*
Symptomatic (*n* = 63)	80.0 ± 10.8	87.7 ± 9.0	*<0.001*
AIDS (*n* = 14)	80.5 ± 12.0	83.7 ± 7.9	*0.38*
HIV services provider			
Hospital (*n* = 235)	82.8 ± 10.4	88.0 ± 9.3	*<0.001*
Community based (*n* = 116)	82.9 ± 11.4	85.5 ± 8.6	*0.03*
Marital status			
Alone (*n* = 194)	82.2 ± 11.1	86.6 ± 9.2	*<0.001*
In couple (*n* = 157)	83.7 ± 10.3	88.0 ± 9.0	*0.001*
HAART status			
No treatment (*n* = 83)	82.0 ± 9.8	81.4 ± 10.7	*0.62*
Under treatment (*n* = 266)	83.2 ± 11.0	89.1 ± 7.8	*<0.001*
Sexual behavior risk			
No risk (*n* = 214)	83.1 ± 10.9	87.4 ± 9.4	*<0.001*
Risk (*n* = 134)	82.6 ± 10.6	86.8 ± 8.7	*<0.001*

^a^Wilcoxon test used

In a logistic regression, a global quality of life score >77 was strongly associated with a lesser increase over time (OR:0.06 95 % CI [0.02–0.16]; *P < 0.001*). Although not statistically significant, the longer a patient stayed under HAART, the more he/she experienced an increase of his/her global quality of life score. Having no support from relatives for medical care at baseline was significantly associated with an increase in the global quality of life score over time. In addition, being under HAART was associated with an increased global quality of life score (OR:3.08, 95 % CI [1.59–5.96];*P = 0.001*). Finally, self perception as healthy was also associated with an increased global quality of life score over time (Table 4).

**Table 4:** Factors associated with an increase of the global score of quality of life over 12 months in PLWHA in Ouagadougou, Burkina Faso

	Total	People with increased score of QOL	Univariate Unadjusted	Multivariate Adjusted	P for Adjusted OR
	n^a^	n (%)	OR (95 % CI)	OR (95 % CI)	
Gender					
Man	42	21(50.0)	1		
Woman	298	193(64.8)	1.83(0.96–3.52)		
Support for medical care					
No	134	92(68.7)	1	1	
Yes	205	121(59.0)	0.65(0.41–1.04)	0.56(0.32–0.97)	*0.04*
Highly Active Antiretroviral Treatment status					
No	80	39(48.8)	1	1	
Yes	258	174(67.4)	2.17(1.30–3.62)	3.08(1.59–5.96)	*0.001*
School					
No	112	79(70.5)	1		
Yes	228	135(59.2)	0.60(0.37–0.98)		
Self perceived as healthy					
No	198	111(56.1)	1	1	
Yes	110	80(72.7)	2.09(1.26–3.46)	1.90(1.05–3.43)	*0.03*
Global score of QOL at baseline					
< =77	90	84(93.3)	1	1	
>77	250	130(52.0)	0.07(0.03–0.18)	0.06(0.02–0.16)	*<0.001*
Treatment group at baseline					
No Treatment	80	40(50.0)	1		
Less than 3 months	19	12(63.2)	1.71(0.61–4.80)		
1 year or more	241	162(67.2)	2.05(1.22–3.42)		

^a^Total may differ accordingly

## Discussion

At baseline, the highest scores were recorded in the domain of spirituality, religion and personal beliefs (SRPB) and the lowest scores were seen in the environmental domain. The environmental domain includes aspects related to the home environment, financial resources, accessibility to health and social care, physical environment and recreational activities. Burkina Faso is a country where 46.5 % of people live below the national poverty line [10]. The socio-economic profile of the country and the professional profile of our respondents dominated by the informal sector and small business could justify those low scores recorded in the environmental domain. In previous studies, similar results with lowest scores in environmental domain and highest scores in SRPB domain were also found in Burkina Faso [5] and in Ethiopia [13, 14]. At the third interview, the lowest and highest scores remained respectively in the environmental domain and in the spiritual domain. These results are consistent with what was found at baseline. Over the 12 months, the fact that the religious practices of the respondents and their environments did not change could justify the sameness of scores in these domains of quality of life. Other previous longitudinal studies have shown the same trends. In a study looking at HIV-Tuberculosis co-infected patients, the spirituality domain showed the highest scores and the environmental domain held the lowest scores after a 6-month follow-up [14]. In the same study, patients with HIV but not co-infected with tuberculosis showed the same trends at the end of the 6 month follow-up.

In our study, four domains of quality of life showed progression at month 6 and at month 12 when comparing to baseline. These domains were the psychological domain, level of independence, social relationships and the environmental domain. These increased scores could be partly explained by the support that PLWHA benefited from in the HIV-care facilities, where our investigation was conducted. These facilities offer, among others, support groups such as psychological and social support, where PLWHA come together and support one another in dealing with their HIV status [15, 16]. Even if the environmental domain recorded increased scores, it remained, over time, the domain with the lowest scores. In our study, the physical domain, the religion, spirituality and personal beliefs domain, showed a sawtooth pattern. Deribew et al., on the other hand, showed a significant increase in all domains of quality of life after a 6 month follow-up of PLWHA initiating HAART [14]. According to HAART status, our respondents under treatment for at least 1 year showed a steadily rising global quality of life score over 12 months. In patients who started treatment at the beginning of our study, the global quality of life score, after undergoing a jump in the sixth month, showed a relative decline, subsequently leading to stabilization. This trend can be explained by the national context and the issues PLWHA are facing. Usually, people in need of treatment in Burkina Faso wait a long time before treatment becomes available, due to financial limits. Starting treatment appears to be a solace for them. This situation, in addition to the physiological benefits of HAART, can justify the leap made in the sixth month in HIV patients who started the treatment at the beginning of the study. In their longitudinal study, Solomon et al. showed how quality of life increased, even if the increase diminished with time, with best increase which occurred between baseline and the sixth month of their 12-month follow-up [17].

Our study showed that being under HAART was associated with an increased global quality of life score. In addition, the longer a patient stayed under HAART, the more he/she experienced an increase of his/her global quality of life score. The fact that people under HAART experienced a greater increase in their global score is consistent with what was found by Liu et al. [18]. Their study showed that HAART was associated with a short term (6 month) improvement in the summary score of quality of life using the Medical Outcome Study (MOS)-HIV. But, they also showed that there was no evidence that HAART modifies trends in a long term follow-up.

Although not statistically significant, better increase was observed in women. In line with gender, Jaquet et al. in their study in Burkina Faso, showed that an increase in Mental Health Status score was significant in women and not in men [6].

In our study, having no support from relatives for medical care was associated with an increase in the global quality of life score. Because HIV is still considered as a family shame, living with HIV puts people on the margins of society or their family. In a previous study in Burkina Faso, Ouedraogo et al. showed that 57.5 % of PLWHA were living without any financial support from families [19]. All facilities where our follow-up was conducted offer the following services to PLWHA: anonymous voluntary counseling and testing (VCT), prevention counseling, medical treatment and monitoring, nutrition counseling, psychological and social support. Among other specific services offered, there is also medication, medical care for opportunist infections, home visits, support groups, and food. These specific activities undertaken by professionals or by peer educators provide those with no family support with another option. The support received from the facilities can explain why, despite having no support from relatives for medical care, the global quality of life score grew, even if it stayed lower than the global score of those who have support from families for their medical care. Psychological support received from HIV facilities may explain the association between self perception as healthy and increased global quality of life score.

In a large study involving four southern African countries, people with lower education reported higher scores of satisfaction with life [20]. Such a result is in line with our study, where illiterate people recorded better proportion with increased score, although not statistically significant.

Studies on the quality of life of persons living with HIV in developing settings have shown the association between, on one hand, the quality of life and, on the other, biological tests, in particular viral load and CD4 cell count [21–23]. In our study, it was not possible to analyze such association because very few patients have updated biological data in their routine follow-up. We assessed quality of life of persons living with HIV in their routine life, which is the daily experience of these people in HIV clinics. Even if the Government of Burkina Faso declared free HAART for those in need since January 1, 2010, PLWHA continue to face problems for getting blood work done (viral load and CD4 count), which are key to monitor the health. While biological monitoring certainly allows for better medical follow-up, it is out of reach for our patients because of the high cost. Some patients manage to do their tests through studies which pay for patients’ laboratory tests. Unfortunately, at the end of such studies, they are faced once again with the cost and discontinued tests. Once research goals are achieved in the context of HIV/AIDS, patients’ medical care is sometimes thwarted by sudden costs. According to national guidelines, these tests have to be done on a 6-month basis [24, 25]. Recent findings showed that annual monitoring of CD4 instead of every 6 months in persons with a CD4 above 250 cells/mm^3^ was sufficient to detect any clinical problem early enough [26]. Such results, associated with the fact that new guidelines suggest starting HAART when CD4 reaches 500 cells/mm^3^ [1], instead of previously indicated levels of 250 or 350 cells/mm^3^, will probably reduce the frequency of CD4 tests in PLWHA in their routine follow-up. As a result, significant cost savings may be made by PLWHA, giving them more opportunity to do this key test.

In our study, about 17 % of patients were lost to follow-up. Most of them were not under HAART. This can be explained by the fact that when people are not under treatment in a facility, they tend to leave that facility and look for another one in which they can find treatment. Such patients, considered as lost to follow-up in our sample, are probably in other facilities, except for two of them who were known to have died during the follow-up and one who openly refused to continue the study. The proportion of patients lost to follow-up was lower than what was recorded by Solomon et al. in their study, with 61.8 % of lost to follow-up [17].

The internal consistency of the scale used in our study gave a Cronbach’s alpha set at 0.85. This value is higher than values usually recommended (ranging from 0.7 to 0.8) [27, 28], which means our scale has good reliability. In a study conducted in South Africa using the same scale we used, the Cronbach’ alpha found was 0.88, which is near to ours [29].

According to the latest data in the general population, the prevalence of HIV is not statistically different by gender (1.2 % in women and 0.8 % in men from 15 to 49 years old) [30]. Our study was comprised of 87.5 % women. According to the data from the Ministry of Health, people living with HIV attending HIV facilities in the country were 69.7 % women [31]. In a previous study in Burkina Faso, a high proportion (72 %), but lower than ours, was recorded, even when the prevalence of HIV was higher in men than in women [32]. The proportion of women in our study is therefore partly explained by the profile of PLWHA attending HIV structures of the city, dominated by women.

### Limitations

Our study was conducted in Ouagadougou, which is not representative of the whole country. However, the facilities where the study was conducted may be a good portrayal of the urban reality of the country. Our sample was based on a systematic sampling method, using the daily queue for routine follow-up in each facility. The number of interviewees per structure was reached after a 1-month enrolment period. Given enrolment was over such a short period, our sample may not be representative of patients in the HIV facilities of the city all year round. Moreover, the proportion of women in our sample, which was higher than what had been found elsewhere in other studies, can limit the representativeness of our sample. Our study did not use biological tests. Nevertheless, an intervention with biological follow-up based on laboratory tests could have been useful for the relationship between quality of life, items such as CD4 count and viral load, and clinical status.

## Conclusions

Our findings suggest conducting interventions linked to the environmental domain to enhance the quality of life of persons living with HIV/AIDS in Burkina Faso. Such interventions should be directed towards empowering persons living with HIV instead of making them dependent on ad hoc support. Particular attention could be paid to women, illiterate people, people who perceive themselves as ill, symptomatic patients and those not yet under treatment. A pilot initiative to make affordable loans available to PLWHA to enable them to start income generating activities was initiated by the Support Programme to Associative and Community World (PAMAC) in Burkina Faso [33]. This operation, with a 98 % repayment rate, has enabled 783 persons living with HIV to have access to these micro credits. The results showed that these people thrived and found a better position in society. They thus manage to provide for their households (health expenses, food, schooling for children, etc.), but are also able to participate in the management of social and family events (weddings, baptisms, funerals, etc.), which constitutes a mark of social integration. Another pilot project through small home gardening or collective gardening initiated in Ouagadougou showed how food needs of PLWHA where met with products of these gardens. The surplus of production was then sold to the population and profits of the sale were used to meet other needs of PLWHA. Extending these two initiatives to a larger number of PLWHA in rural and urban areas will help them meet their basic needs, improve their life and health-related quality of life, and will contribute to job creation. However, such initiatives need to be undertaken through community-based associations fighting against HIV/AIDS for good guidance and a good monitoring.

## Abbreviations

AAS: Association African Solidarity
ALAVI: Association Laafi la Viim
CD4: Cluster of Differentiation 4
CICDoc: Center for Information, Counseling and Documentation on AIDS and Tuberculosis
DHB: District Hospital of Boulmiougou
HAART: Highly Active Antiretroviral Treatment
HIV/AIDS: Human Immunodeficiency Virus/Acquired Immunodeficiency Syndrome
PLWHA: Person Living With HIV/AIDS
QOL: Quality of Life
SRPB: Spirituality, Religion and Personal Beliefs
TB: Tubercle Bacillus (Tuberculosis)
UHYO: University Hospital Yalgado Ouédraogo
WHOQOL-HIV: World Health Organization Quality of Life assessment scale in patients with Human Immunodeficiency Virus infection
WHOQOL HIV-BREF: World Health Organization Quality of Life assessment brief scale in patients with Human Immunodeficiency Virus infection
